# BDNF Acts as a Prognostic Factor Associated with Tumor-Infiltrating Th2 Cells in Pancreatic Adenocarcinoma

**DOI:** 10.1155/2021/7842035

**Published:** 2021-11-05

**Authors:** Yanan Zhu, Cangang Zhang, Dongyu Zhao, Wenhua Li, Zhe Zhao, Shukun Yao, Dongyan Zhao

**Affiliations:** ^1^Department of Pathogenic Microbiology and Immunology, School of Basic Medical Sciences, Xi'an Jiaotong University, Xi'an, China; ^2^Department of Student Affairs, Puyang Medical College, Puyang, China; ^3^College of Life Science, Northwest University, Xi'an, China; ^4^Graduate School, Chinese Academy of Medical Sciences and Peking Union Medical College, Beijing, China; ^5^Department of Gastroenterology, China-Japan Friendship Hospital, Beijing, China

## Abstract

Pancreatic adenocarcinoma (PAAD) is an extremely lethal disease worldwide. Brain-derived neurotrophic factor (BDNF) is a critical member of the neurotrophin polypeptide superfamily that plays an important role in multiple cancers. However, the association among BDNF expression, tumor immunity, and PAAD prognosis remains unclear. BDNF expression and its influence on patient prognosis were explored based on The Cancer Genome Atlas, Cancer Cell Line Encyclopedia, Genotype-Tissue Expression, and Kaplan-Meier plotter. Gene set enrichment analysis was performed to understand the biological roles of BDNF. The role of BDNF in tumor-infiltrating immune cells was determined using the Tumor Immune Estimation Resource database and the single-sample gene set enrichment analysis and xCell algorithm. The correlation among BDNF and chemokines, chemokine receptors, chemotherapeutic efficacy, and immune checkpoints was analyzed based on RStudio. BDNF expression was remarkably higher in PAAD compared to their paired normal tissues, and high BDNF expression was associated with unfavorable prognosis. Enrichment analysis revealed that BDNF was significantly enriched in major oncogenic pathways in PAAD. BDNF expression was positively correlated with immune infiltration, especially Th2 cells. Moreover, BDNF expression was positively correlated with Th2 cell-related chemokine/chemokine receptors, indicating that BDNF might modulate the migration of Th2 cells in PAAD. We also found that BDNF expression was correlated with high chemotherapeutics sensitivity and highly expressed immune checkpoints, making it a valuable biomarker in predicting the therapeutic benefits for chemotherapy and immunotherapy in cancer patients. In summary, BDNF might affect patient prognosis by interacting with tumor-infiltrating Th2 cells, thus serving as a potential prognostic biomarker in PAAD.

## 1. Introduction

Pancreatic adenocarcinoma (PAAD) is an extremely lethal disease found in the digestive system, ranking as the seventh leading cause of cancer-related death in both men and women worldwide [1]. Despite the morbidity and mortality rates of other malignant tumors gradually declining, the morbidity rates of and number of cancer-related deaths caused by PAAD are still increasing [2]. Due to the indistinct symptoms of early-stage PAAD, rapid tumor progression, and limited efficacy of early diagnostic methods, the majority of PAAD patients present with unresectable or metastatic tumors, with their 5-year survival rates less than 10% [2]. Multiagent conventional chemotherapy regimens are the mainstay of treatment for individuals diagnosed with advanced or metastatic PAAD, providing only months of overall survival (OS) benefit [3–5]. Such unfavorable clinical outcomes have fueled ongoing efforts to explore the complex feature of the tumor microenvironment (TME) and the molecular mechanisms underlying pancreatic carcinogenesis, and evidence has recently shown that immunotherapy targeting the interaction between tumor-infiltrating lymphocytes and tumor cells has been proposed as a promising therapy for PAAD [6, 7]. The use of immune checkpoint blockade has demonstrated robust results in many malignancies, but unfortunately, it is not yet translated to PAAD, possibly attributing to the immunosuppressive TME [7–10]. Accumulating evidence has demonstrated that immune cells, the key component of TME, are of particular relevance in patient prognosis and the antitumor efficacy of chemotherapy and immunotherapy [11, 12]. Therefore, it is highly important to elucidate the complicated and multifaceted roles of the complex TME components in pancreatic carcinogenesis and progression and identify novel therapeutic biomarkers associated with tumor infiltration in PAAD.

Brain-derived neurotrophic factor (BDNF), a member of neurotrophins (NTs) originally known for its well-documented functions on the development and regeneration of central nervous system, has aroused attention because of its critical roles in human malignancies [13]. BDNF and its high-affinity tyrosine kinase receptor, tropomyosin receptor kinase B (TrkB), have been demonstrated to be overexpressed in multiple cancers, such as neuroblastoma, oral, breast, lung, and colorectal cancer. Moreover, its upregulated expression is proven to be remarkably correlated with adverse clinical outcomes [14–18]. There is now extensive literature demonstrating that BDNF/TrkB signaling is involved in regulating several aspects of tumor cell physiology, including neovascularization [19], tumor proliferation [20], epithelial-to-mesenchymal transition [21], apoptosis resistance [22], and cytotoxic drug resistance [23]. Thus far, a detailed understanding of the precise mechanisms and functions of BDNF in PAAD is lacking. Intriguingly, BDNF has been reported to be related to immune modulation in neurogenic and nonneurogenic tissues. For example, BDNF can regulate local inflammation by modulating the level of transcription factors and cellular cytokines in brain tissues of patients suffering from ischemic stroke [24]. Xiao et al. have shown that environmental and genetic activation of hypothalamic BDNF could lead to a decreased peripheral CD4 : CD8 ratio, whereas knockdown of hypothalamic BDNF could eliminate the change [25]. Regarding nonneurogenic tissues, BDNF is implicated in T cell maturation, B cell survival, and eosinophilic granulocyte activation, implying that BDNF is a potential factor in modulating immune homeostasis [26–28]. However, there is limiting evidence regarding the BDNF-correlated functions in tumor immunology, and more comprehensive analysis of BDNF profile in PAAD is expected to understand the precise functions of BDNF in prognosis and tumor immune infiltration.

Advancements in sequencing technologies largely enhance our understanding of cancer genomics, making it possible to explore the molecular mechanisms of multiple carcinomas through bioinformatics analysis in publicly available genomic datasets. Thus, this study is broadly aimed at studying the mechanisms underpinning the oncogenic role of BDNF in PAAD by combining available information from several public databases, including Cancer Cell Line Encyclopedia (CCLE), The Cancer Genome Atlas (TCGA), Gene Expression Omnibus (GEO), Genotype-Tissue Expression (GTEx), International Cancer Genome Consortium (ICGC), Tumor Immune Estimation Resource (TIMER), Gene Expression Profiling Interactive Analysis (GEPIA), and Kaplan-Meier plotter. First, the profile of BDNF was estimated in human cancer cell lines, human normal tissues, and multiple cancer types. Subsequently, the associations between BDNF expression and patients' prognosis and clinicopathological characteristics were evaluated based on the TCGA database and Kaplan-Meier plotter. Identification of similar genes correlated with BDNF and gene set enrichment analysis were applied to understand the biological roles of BDNF in PAAD. Subsequently, the role of BDNF in tumor-infiltrating immune cells was determined using the TIMER database and the single-sample gene set enrichment analysis (ssGSEA) and xCell algorithm. Finally, we estimated the correlation among BDNF and chemokines, chemokine receptors, chemotherapeutic efficacy, and immune checkpoints.

## 2. Materials and Methods

### 2.1. BDNF Gene Expression Analysis

Gene expression data were downloaded from the four publicly available datasets, CCLE database, TCGA database, GEO database, GTEx project, and ICGC database. Missing and duplicated results were removed from the preprocessed data, which were subsequently normalized by log2(TPM + 1) based on the “rma” function in affy (v1.68) package. Gene expression data in normal tissues were extracted from the GTEx project and were used to compare the mRNA levels of BDNF among different tissues/organs. The CCLE dataset, a publicly available genomic dataset that includes gene expression profiles from 917 human cancer cell lines spanning 36 cancer types, was used to present the distribution of BDNF expression in different types of human cancer cell lines from various organizations [29]. The Kruskal-Wallis test was conducted to determine the differences among organs. Moreover, gene expression profiles from the GTEx and TCGA project were utilized to investigate BDNF expression across different cancer types compared to TCGA and GTEx normal tissues.

### 2.2. Prognosis Analysis of BDNF in PAAD

From the TCGA-PAAD dataset, gene mRNA expression data and the corresponding clinical information from 179 tumor tissues and 4 paracancerous tissues were downloaded, in which the acquisition and application procedures aligned to the protocol. PAAD patients were firstly divided into the high and low expression subgroups based on the median value of the BDNF expression. Afterward, the Kaplan-Meier curves were plotted using the online tool Sangerbox to explore the association between BDNF expression and progression-free survival (PFS). The analysis of overall survival (OS) and relapse-free survival (RFS) between the high and low BDNF expression subgroups in PAAD patients was also performed using the Kaplan-Meier plotter (https://kmplot.com/analysis/), a web-based platform capable of assessing the effectiveness of a wide array of genes on patients' survival rates in 21 cancer types. The survival difference between the high and low BDNF expression subgroups was calculated by the hazard ratios (HRs) and log-rank *p* value.

### 2.3. Correlation Analysis between BDNF and Clinicopathological Features

To explore the association between BDNF expression and clinicopathological characteristics of PAAD patients, patients with complete information on clinicopathological variables, including histological grade, age, sex, and American Joint Committee on Cancer (AJCC) stage, were selected from the TCGA database. Subsequently, a series of chi-squared tests were performed to verify the association between BDNF levels and clinicopathological parameters in PAAD, and the R package “ComplexHeatmap” (v2.6.2) was utilized for comprehensive visual presentation. We further used Wilcoxon rank-sum tests to compare the difference in BDNF expression among different subgroups of clinicopathological variables, and these results were presented as box diagrams.

### 2.4. Determination of BDNF Functions in PAAD

Before identifying the biological functions of BDNF, similar genes that have a similar expression pattern with BDNF in PAAD were extracted from the GEPIA database (http://gepia.cancer-pku.cn/). The GEPIA database is a web-based tool for analyzing RNA sequencing expression data and providing customizable functions such as patient survival analysis, correlation analysis, and similar gene detection, which includes 9736 tumors and 8587 normal samples from the TCGA and GTEx data [30]. Pathway enrichment analysis including Gene Ontology (GO) terms and Kyoto Encyclopedia of Genes and Genomes (KEGG) pathways was performed using the “clusterProfiler” (v3.18.0) package and “org.Hs.eg.db” (v3.12.0) package to elucidate the underlying biological mechanisms of BDNF and its top 100 similar genes. Moreover, the results of GO and KEGG pathway analyses were considered to indicate significance at a cut-off threshold of *p* < 0.05 and *q* < 0.05. To further validate the enrichment results, the associations between BDNF and tumor-associated genes in PAAD were analyzed using Pearson correlation analysis based on the TCGA data. The “ggplot2” (v3.3.2) package in R software was applied to visualize the enrichment results to help interpret these results.

### 2.5. Correlation Analysis between BDNF and Immune-Infiltrating Cells

The association between BDNF expression and tumor-infiltrating immune cells was determined using the ssGSEA algorithm. ssGSEA is a rank-based algorithm that requires an input matrix of gene signatures expressed by immune cell populations and accurately determines the relative immune cell infiltration levels of individual cancer samples [31]. Based on RNA-Seq expression data, the R package “gsva” (v1.38.0) was utilized to quantify the relative levels of immune cells in each patient included in the TCGA project, and Pearson's correlation was employed to assess how BDNF expression correlated with the levels of infiltrating immune cell subtypes.

### 2.6. Protein-Protein Interaction Network Analysis

A protein-protein interaction (PPI) network was constructed based on top 100 similar genes that significantly correlated with BDNF by using Search Tool for the Retrieval of Interacting Genes (STRING, version 11.5, http://string-db.org) database. STRING is an online tool used to predict the functional interactions between proteins, which is essential for recognizing the mechanisms of cell activities at the molecular levels in cancer progression. The disconnected nodes were hided in the network. Then, the PPI network was visualized by the Cytoscape software (version 3.7.2, http://www.cytoscape.org/).

### 2.7. Validating the Association between BDNF and Th2 Cells

TIMER2.0 (https://cistrome.shinyapps.io/timer/) is a web-based data-mining platform that includes 10,897 samples across 32 cancer types and applies multiple immune deconvolution methods to determine the relative levels of immune infiltrates from their gene expression profiles [32]. We initially employed the TIMER2.0 database to explore the association between BDNF gene expression and the levels of Th2 cell infiltration. The correlations between BDNF expression and the gene markers for Th2 cells were also assessed using the TIMER2.0 database. The gene markers of Th2 cells were selected from the website of R&D Systems (https://www.rndsystems.com/cn/resources/cell-markers/immune-cells), and the Spearman method was used to determine the correlation coefficient. Furthermore, we performed further OS analysis for combining the mRNA levels of BDNF and different abundances of Th2 cells in PAAD patients.

Two independent human PAAD datasets obtained from publicly available genomic datasets were used as external validation to further explore the impact of BDNF on the abundance of Th2 cells in PAAD patients: GSE85916 and ICGC PAAD-AU cohort. Original Series Matrix Files of GSE85916 were collected from the Gene Expression Omnibus database (https://www.ncbi.nlm.nih.gov/geo/). GSE85916 was submitted by Puleo F and colleagues and contained 80 PAAD tissues. Australian pancreatic cancer RNA-sequencing data (ICGC PAAD-AU cohort) encompassing 242 samples were retrieved from the ICGC (https://dcc.icgc.org/releases). The “xCell” (v1.1.0) package, a high-resolution gene signature-based method that can generate a reliable score for up to 64 immune and stromal cell types, was employed to calculate the cell type enrichment scores of Th2 cell infiltration for each PAAD sample based on the gene expression data [33]. Afterward, Wilcoxon rank-sum tests were conducted to compare the difference in Th2 cell infiltration between the high and low BDNF expression subgroups in the TME of PAAD patients.

### 2.8. Exploration of the Significance of BDNF in Clinical Treatment

To explore the role of BDNF in the medicinal therapy for PAAD patients, drug sensitivity (lower half inhibitory concentration [IC50]) in response to common anticancer drugs such as paclitaxel, gemcitabine, rapamycin, obatoclax mesylate, AKT inhibitor VIII, and c-Jun N-terminal kinase (JNK) inhibitor VIII was calculated in the TCGA-PAAD dataset. Next, the difference in drug sensitivity between the low and high BDNF expression subgroups was compared using the Wilcoxon rank-sum tests and visualized with the R package “pRRophetic” (v0.5) and “ggplot2” (v3.3.2). In addition, we performed a series of Pearson correlation analyses to investigate the association between BDNF expression and gene expression levels of immune checkpoints (including but not limited to PDCD1, CD274, and CTLA4) and chemokines and chemokine receptors based on the TCGA-PAAD dataset.

### 2.9. Statistical Analyses

R software (version 3.6.1) was employed to implement the statistical analyses in the study. *p* values < 0.05 were considered significant unless otherwise specified.

## 3. Results

### 3.1. Expression Levels of BDNF in Pan-Cancer

**Figure 1:**
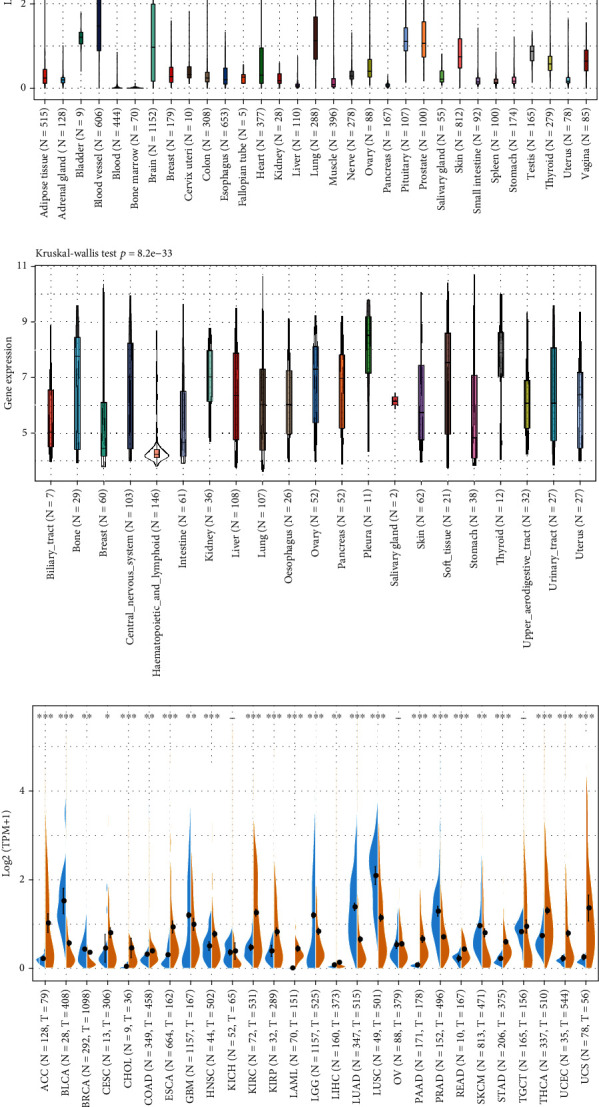
BDNF mRNA expression in GTEx normal tissues, human cancer cell lines, and TCGA cancer tissues. (a) BDNF mRNA expression in GTEx normal tissues. (b) BDNF mRNA expression in various tumor cell lines, analyzed by the CCLE database. (c) Expression level of BDNF across 27 TCGA tumors compared to TCGA normal and GTEx normal tissues. The key to all TCGA abbreviations is shown in Supplementary Table 1. ^∗^*p* < 0.05, ^∗∗^*p* < 0.01, ^∗∗∗^*p* < 0.001. GTEx: Genotype-Tissue Expression; TCGA: The Cancer Genome Atlas. CCLE: Cancer Cell Line Encyclopedia.

To achieve a better understanding of the role of BDNF in cancers, the distribution of BDNF in various types of normal and tumor tissues was analyzed using gene expression data from the GTEx, CCLE, and TCGA projects. Using data from the GTEx database comprising 31 normal tissues from healthy people, BDNF was proven to be expressed at a low level across most of the organs. Low BDNF expression was observed in the blood, bone marrow, liver, and pancreas, whereas the relatively high expression of BDNF was noted in the blood vessel, bladder, and prostate (Figure 1(a)). The Kruskal-Wallis test indicated that there were significant differences in BDNF expression among the normal tissues (Kruskal-Wallis test *p* = 0). Subsequently, we analyzed the gene expression profiles of BDNF in nearly 1000 human cancer cell lines extracted from the CCLE dataset, and the results revealed that the mRNA expression levels of BDNF not only were increased ubiquitously in contrast to the range of expression in the normal tissues but also varied significantly among different cancer cell lines (Kruskal-Wallis test *p* = 8.2*e* − 33, Figure 1(b)). High mRNA expression of BDNF was noted in many cancer lines, especially those originating from the bone, pancreas, and pleura, whereas the relatively low expression was observed in the breast, intestine, and hematopoietic and lymphoid organs.

To confirm the above observation, we further plotted BDNF expression distribution across multiple TCGA cancer types and normal tissues. Considering that the number size of normal samples in the TCGA database was significantly small, we merged the data of normal samples in the GTEx portal with the data of adjacent noncancerous tissues in the TCGA dataset for pan-cancer analyses. As depicted in Figure 1(c), the difference of BDNF expression between tumor and normal tissues achieved significance in 24 out of 27 cancer types, with the evident exception of kidney chromophobe (KICH), ovarian serous cystadenocarcinoma, and testicular germ cell tumor. BDNF expression was more upregulated in many cancers compared to normal tissues, including thyroid, ovary, liver, colorectal, gastric, kidney, esophageal, and pancreatic cancers and acute myeloid leukemia. However, skin, lung, prostate, breast, and brain tumors showed the reverse results with significance. Taken together with the results of CCLE and TCGA pan-cancer analysis, BDNF was commonly overexpressed in digestive cancers such as esophageal, stomach, colon, and pancreatic cancers, which indicated that BDNF may serve as a potential regulatory mechanism in tumor progression and patient prognosis.

### 3.2. Prognostic Potential of BDNF in PAAD

**Figure 2:**
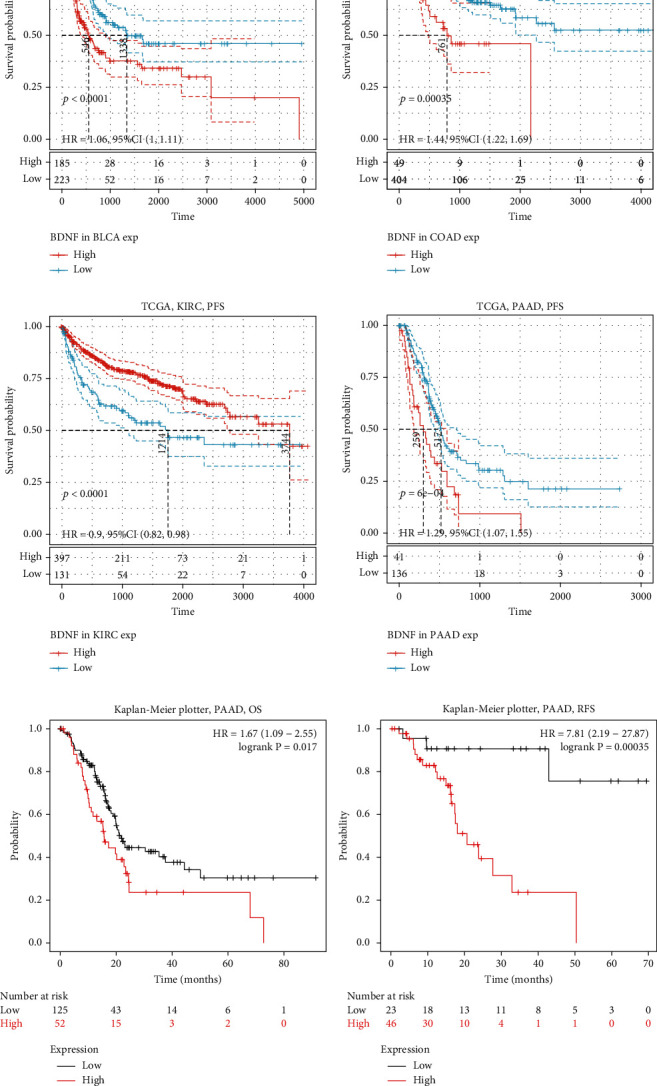
The Kaplan-Meier survival curve analysis of the prognostic significance of high and low expression of BDNF in different cancer types. (a) Kaplan-Meier survival curves comparing the high and low expression of BDNF in different cancer types using TCGA dataset. (b) Kaplan-Meier survival curves comparing the high and low expression of BDNF in PAAD using the Kaplan-Meier plotter. TCGA: The Cancer Genome Atlas; PFS: progression-free survival; OS: overall survival; RFS: relapse-free survival.

Next, we investigated the prognostic value of BDNF for cancer patients in different databases. We found that multiple cancer types exhibited a significant association between BDNF expression and PFS in the TCGA database, including bladder, colon, kidney, and pancreatic cancers (Figure 2(a)). Notably, PFS analysis revealed that BDNF played a protective role in kidney renal clear cell carcinoma (KIRC) (PFS: HR = 0.90, 95%confidence interval [CI] = 0.82–0.98, *p* < 0.001). However, BDNF played a detrimental role in bladder urothelial carcinoma (PFS: HR = 1.06, 95%CI = 1.00–1.11, *p* < 0.001), colon adenocarcinoma (PFS: HR = 1.44, 95%CI = 1.22–1.69, *p* < 0.001), and PAAD (PFS: HR = 1.29, 95%CI = 1.07–1.55, *p* < 0.001). Thus, we focused on PAAD in further study. To further identify BDNF as an adverse prognostic factor for PAAD patients, the Kaplan-Meier plotter database was additionally employed to assess the effect of BDNF expression on OS and RFS. Considering the results derived from the TCGA database, the poorer prognosis was significantly associated with the elevation of BDNF expression in PAAD patients (OS: HR = 1.69, 95%CI = 1.09–2.55, *p* = 0.017; RFS : HR = 7.81, 95%CI = 2.19–27.87, *p* < 0.001; Figure 2(b)). These results confirmed the notion the BDNF expression has an impact on PAAD prognosis.

### 3.3. Associations between BDNF and Clinicopathological Parameters of PAAD Patients

**Figure 3:**
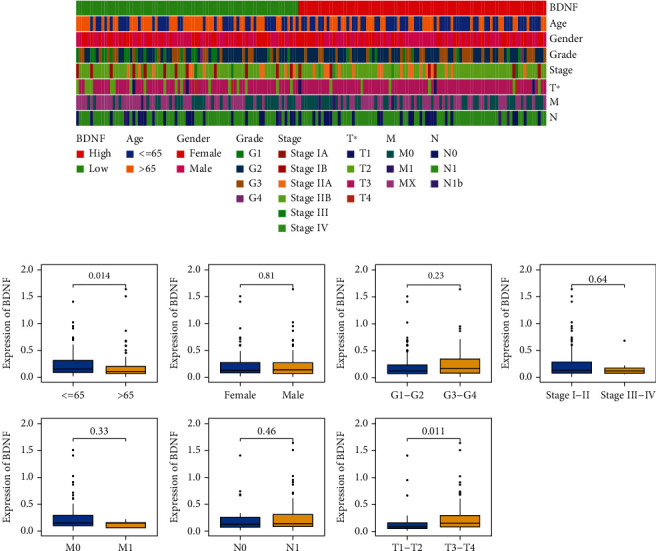
Correlation between BDNF mRNA expression and clinical characteristics of PAAD patients from TCGA database. (a) A strip chart showed that T stage was significantly associated with BDNF expression. (b) Scatter diagrams showed that T stage and age were significantly associated with BDNF expression. ^∗^*p* < 0.05; ns: not significant; TCGA: The Cancer Genome Atlas.

The correlation between BDNF levels and clinicopathological parameters of PAAD patients was assessed using two methods, including the chi-squared and Wilcoxon rank-sum tests. The strip chart obtained by the chi-squared tests showed that T stage was significantly associated with BDNF expression (Figure 3(a)). Scatter diagrams obtained by Wilcoxon rank-sum tests demonstrated that high levels of BDNF were observed in PAAD patients with the following characteristics (Figure 3(b)): aged less than 65 years (*p* = 0.014) and AJCC T3–T4 stage (*p* = 0.011). However, there were no significant differences observed in the correlations of BDNF expression with sex (*p* = 0.81), AJCC stage (*p* = 0.64), AJCC M stage (*p* = 0.33), AJCC N stage (*p* = 0.46), or histological grade (*p* = 0.23).

### 3.4. Functional Enrichment Analyses of BDNF in PAAD

**Figure 4:**
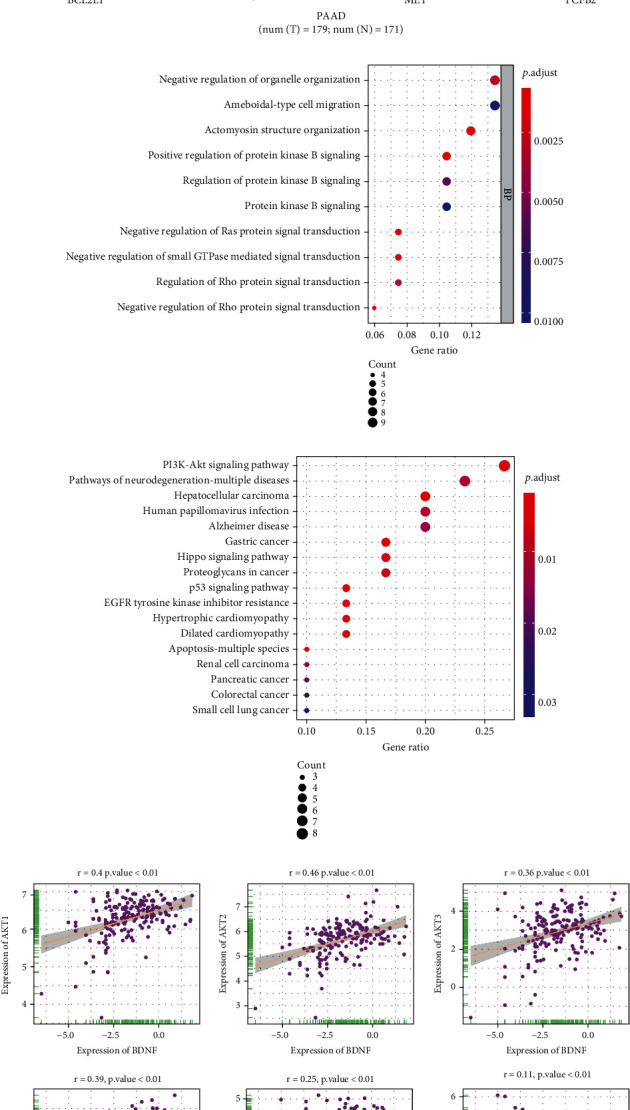
Functional enrichment analysis of BDNF and its similar genes. (a) A PPI network based on top 100 similar genes that significantly correlated with BDNF using Cytoscape. (b) Expression level of four genes extracted from PPI network in PAAD tissues compared to normal tissues in TCGA datasets. (c) The Kyoto Encyclopedia of Genes and Genomes pathways in enrichment analysis of BDNF and its similar genes; (d) top 10 of gene ontology enrichment analysis results of BDNF and its similar genes. Bubble color refers to the enrichment *p* value, and the size of the bubble represents the gene number. (e) Correlation analysis between BDNF and tumor-associated genes in PAAD. PPI: protein-protein interaction.

To further clarify the underlying roles of BDNF in PAAD, we firstly identified the top 100 similar genes that significantly correlated with BDNF using the GEPIA database (Supplementary Table 2). Then, these top 100 similar genes were analyzed in the STRING database, and a PPI network was constructed by the Cytoscape (Figure 4(a)). We found BCL2L1, IRS1, MET, and TGFB2 interacted with BDNF directly. Notably, these genes were highly expressed in PAAD tissues compared with normal tissues, suggesting that BDNF might exert its tumor-promoting effect through these four genes in pancreatic tumor (Figure 4(b)). Furthermore, the biological functions of BDNF and its similar genes were determined by the KEGG pathway and GO annotation analyses, and the results demonstrated that 17 KEGG pathways (Supplementary Table 3) and 30 GO terms (Supplementary Table 4) were enriched for these genes (*p* value < 0.05 and *q* value < 0.05). KEGG enrichment analysis demonstrated significant enrichment of multiple cancers, such as hepatocellular carcinoma, gastric cancer, renal cell carcinoma, pancreatic cancer, colorectal cancer, and small cell lung cancer, which further validated the pan-cancer analysis (Figure 4(c)). Moreover, some of KEGG pathways were observed for their roles in the development and progression of cancer, such as PI3K-Akt signaling pathway, p53 signaling pathway, and Hippo signaling pathway (Figure 4(c)). It was worth noting that BDNF and its similar genes were notably associated with EGFR tyrosine kinase inhibitor resistance, which might provide evidence for their potential role in cancer-targeted treatment. The top 10 terms in the GO results were demonstrated in Figure 4(d). According to GO enrichment analysis, these genes were primarily involved in regulating multiple cancer-promoting signaling pathways, such as protein kinase B signaling, Ras protein signal transduction, and Rho protein signal transduction. All the above results indicated that BDNF and its similar genes could collectively affect tumor-related signaling pathways.

To gain deeper insights into the function of BDNF in the development and progression of pancreatic tumors, we performed correlation analyses to reveal the relevance between BDNF and prooncogenic signaling pathways in PAAD. As depicted in Figure 4(e), BDNF expression was positively correlated with tumor-associated genes, including Akt1, Akt2, Akt3, mTOR, mitogen-activated protein kinase (MAPK) 8, MAPK9, MAPK10, ACTRT3, CDA, BCL2, and MCL1. These tumor-associated genes that seemed to be elevated with BDNF expression level increased. Of note, abnormal expression of BDNF might lead to pancreatic carcinogenesis, resulting in poor survival in PAAD patients.

### 3.5. Associations between BDNF and Immune-Infiltrating Cells

**Figure 5:**
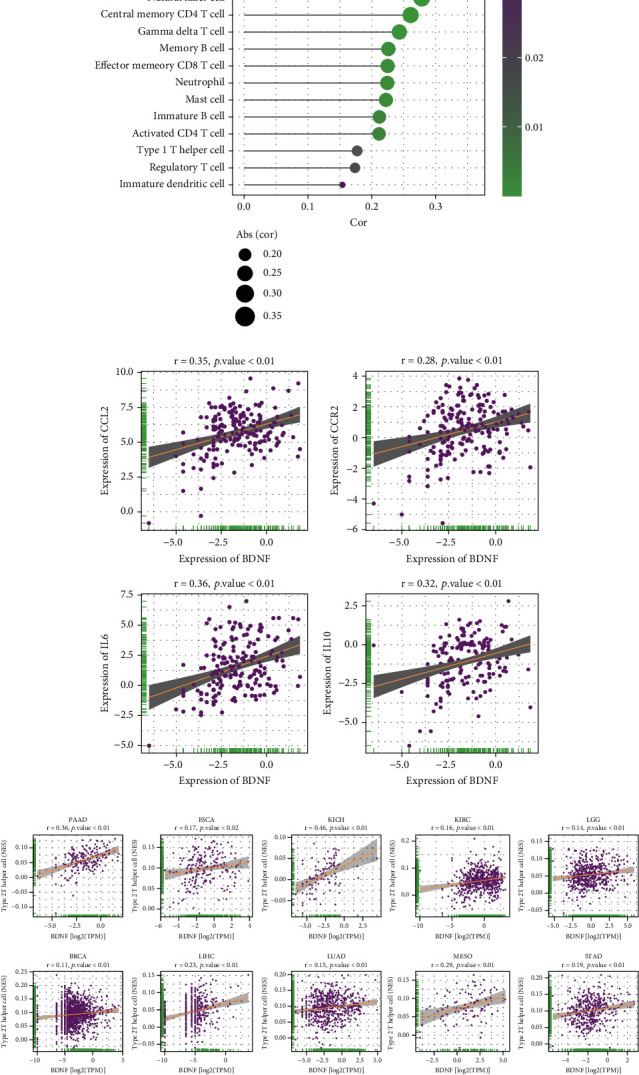
Correlation analysis between BDNF expression and tumor-infiltrating immune cells using the ssGSEA algorithm. (a) Pearson correlations of BDNF expression with the infiltration levels of various types of immune cells (ssGSEA score) in PAAD tissues. (b) Pearson correlations of BDNF expression with the infiltration-related and functional genes of Th2 cells. (c) Pearson correlations of BDNF expression with the infiltration levels of Th2 cells (ssGSEA score) in different cancer types from TCGA dataset. ssGSEA: single-sample gene set enrichment analysis; TCGA: The Cancer Genome Atlas.

It is well known that the survival times of cancer patients can be affected by the quantity and activity status of tumor-infiltrating immune cells in the TME [11, 12]. As the above results revealed that BDNF might play a prognostic role in PAAD, it would be meaningful to analyze the correlation between BDNF expression and immune cells. Thus, we used the ssGSEA algorithm to determine whether BDNF levels were associated with immune cells in PAAD by calculating the Pearson correlation coefficient. These results indicated that BDNF expression had significant correlations with plasmacytoid dendritic cells and multiple types of T cells, including type 2 T helper cell (Th2 cell), central memory CD8+ T cell, natural killer T cell, and central memory CD4+ T cell (Figure 5(a)). Notably, Th2 cells were the immune cell type most strongly associated with BDNF expression in PAAD (*r* = 0.36, *p* < 0.01, Figure 5(a)). BDNF expression was significantly correlated with CCL2-CCR2 (CCL2, *r* = 0.35, *p* < 0.01; CCR2, *r* = 0.28, *p* < 0.01, Figure 5(b)) signaling axis in PAAD, which has been proven to be associated with Th2 polarization and infiltration [34]. Since studies have shown that Th2 cells suppress the antitumor immune response by producing various kinds of cytokines, such as interleukin 6 (IL6) and interleukin 10 (IL10) [35, 36], we further analyzed the relationship of BDNF with IL6 and IL10 expressions in PAAD and found that BDNF was positively associated with IL6 (*r* = 0.36, *p* < 0.01, Figure 5(b)) and IL10 (*r* = 0.32, *p* < 0.01, Figure 5(b)). Moreover, we explored the association between Th2 cells and BDNF expression in multiple cancer types. As shown in Figure 5(c), BDNF expression was also significantly correlated with the infiltration levels of Th2 cells in many cancers, including esophageal carcinoma (*r* = 0.17, *p* = 0.02), KICH (*r* = 0.46, *p* < 0.01), KIRC (*r* = 0.16, *p* < 0.01), brain lower-grade glioma (*r* = 0.14, *p* < 0.01), breast invasive carcinoma (*r* = 0.11, *p* < 0.01), liver hepatocellular carcinoma (*r* = 0.23, *p* < 0.01), lung adenocarcinoma (*r* = 0.15, *p* < 0.01), mesothelioma (*r* = 0.29, *p* < 0.01), and stomach adenocarcinoma (*r* = 0.19, *p* < 0.01). Collectively, these findings supported the notion that BDNF might affect the survival time of cancer patients via interacting with Th2 cells.

### 3.6. Correlation between BDNF and Th2 Cells in PAAD

**Figure 6:**
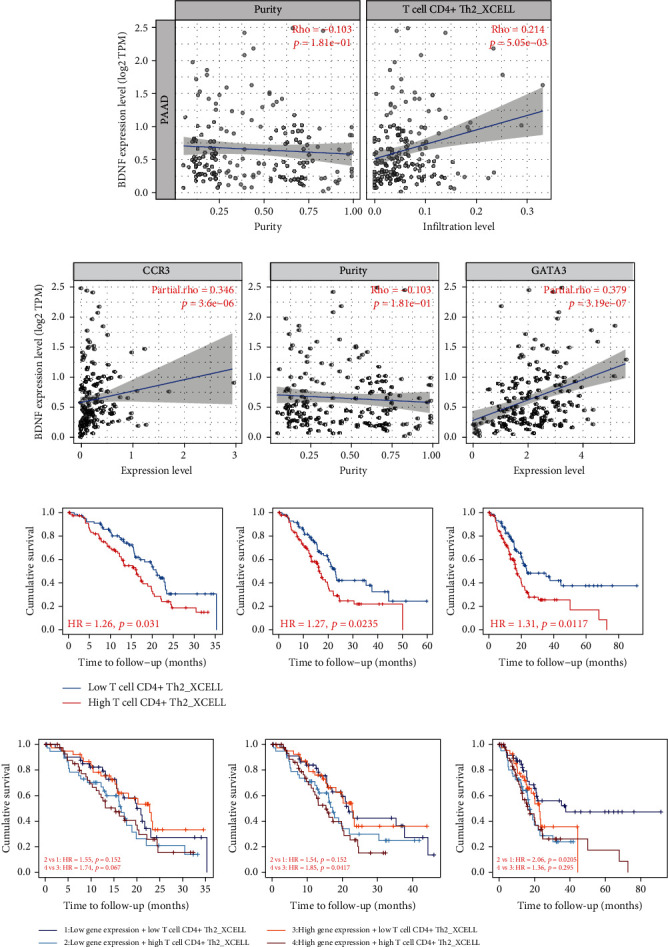
Correlation analysis between BDNF expression and tumor infiltrating Th2 cells in PAAD by using the TIMER database. (a) Spearman correlations of BDNF expression with the infiltration levels of Th2 cells in PAAD tissues. (b) Spearman correlations of BDNF expression with the expression of marker genes of Th2 cells in PAAD tissues. (c) Overall survival analysis for Th2 cells in PAAD patients. (d) Overall survival analysis for combining the expression of BDNF and Th2 cells in PAAD patients.

To further explore the potential associations between BDNF expression and the infiltrating levels of Th2 cells in PAAD, we employed the xCell algorithm to estimate the association between BDNF expression and Th2 cells using the TIMER database. After adjusting the correlation for tumor purity, BDNF expression was remarkably associated with immune infiltration level of Th2 cells in PAAD patients (*r* = 0.214, *p* = 0.005, Figure 6(a)). We also found significant association between BDNF expression and Th2 cell markers, such as CCR3 (*r* = 0.346, *p* < 0.001), STAT6 (*r* = 0.273, *p* < 0.001), and GATA3 (*r* = 0.379, *p* < 0.001, Figure 6(b)). Moreover, due to the importance of Th2 cells in the process of tumor immunosuppression, we further investigated the association between Th2 cells and patients' prognosis in PAAD using the TIMER database. Kaplan-Meier plots demonstrated that high levels of Th2 cells were correlated with not only poorer short-term OS rates but also unfavorable long-term OS rates (Figure 6(c)). We also evaluated the difference of OS among patients stratified by both the estimated infiltration levels of Th2 cells and BDNF expression levels. As illustrated in Figure 6(d), there was no remarkable correlation between Th2 cells and prognosis at short-term follow-up (up to 35 months) when combined with the expression patterns for BDNF. Interestingly, at medium-term follow-up (up to 50 months), lower infiltration levels of Th2 cells predicted favorable prognosis under the high expression levels of BDNF (HR = 1.85, *p* = 0.0417), whereas no significant association between Th2 cells and prognosis was found under the low expression levels of BDNF (HR = 1.54, *p* = 0.152). In contrary with these results, patients with the combination of high Th2 cell levels and low BDNF expression levels experienced worse outcomes at long-term follow-up (up to 100 months) (HR = 2.06, *p* = 0.0205). However, there was no significant correlation between Th2 cells and prognosis under the high BDNF levels at long-term follow-up (HR = 1.36, *p* = 0.295). These results suggested that combining the expression levels of BDNF with Th2 cells might play a vital role in the accurate prediction of prognosis in PAAD patients.

**Figure 7:**
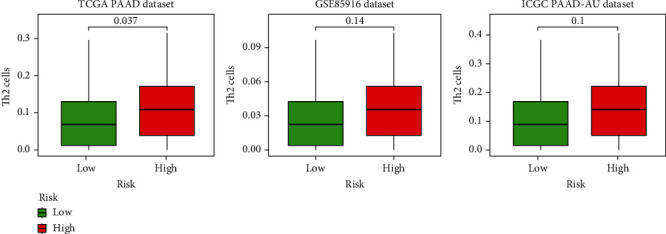
Three independent human PAAD cohorts were used to explore the impact of BDNF on the abundance of tumor-infiltrating Th2 cells.

To further verify the significant differential infiltration levels of Th2 cells in PAAD patients stratified by BDNF expression levels, the TCGA-PAAD cohort and GSE85916 and ICGC PAAD-AU cohort were used to evaluate the levels of Th2 cells in the high BDNF expression and low expression subgroups as internal validation and external validation, respectively. As exhibited in Figure 7, the infiltration levels of Th2 cells in the TCGA cohort were more remarkably upregulated in the high BDNF expression subgroup compared to that in the low expression subgroup (*p* = 0.037). Similar tendencies, albeit not statistically significant levels, were observed in the GSE85916 (*p* = 0.14) and ICGC PAAD-AU cohort (*p* = 0.10).

### 3.7. Associations between BDNF and Chemokines and Chemokine Receptors

To further clarify the potential role of BDNF in cancer immunity, we estimated the correlation of BDNF expression levels with chemokines and chemokine receptors, which were best known for their ability to orchestrate the proper movement of immune cells. As Tables 1 and 2 demonstrated, BDNF expression was positively associated with Th2-associated chemokines and chemokine receptors, including CCL17, CCL22, and CCR4. Moreover, we found that BDNF expression was strongly correlated with CCR2, CXCR2, and CXCR4, and the inhibitors of these chemokine receptors were already evaluated in many preclinical studies and clinical trials for PAAD [37–39]. Most of the chemokines and chemokine receptors tended to be increased accompanied by the upregulation of BDNF expression. Consequently, increased expression of BDNF might lead to the migration of immune cells to tumor tissues.

### 3.8. Exploration of the Significance of BDNF in Clinical Treatment

**Figure 8:**
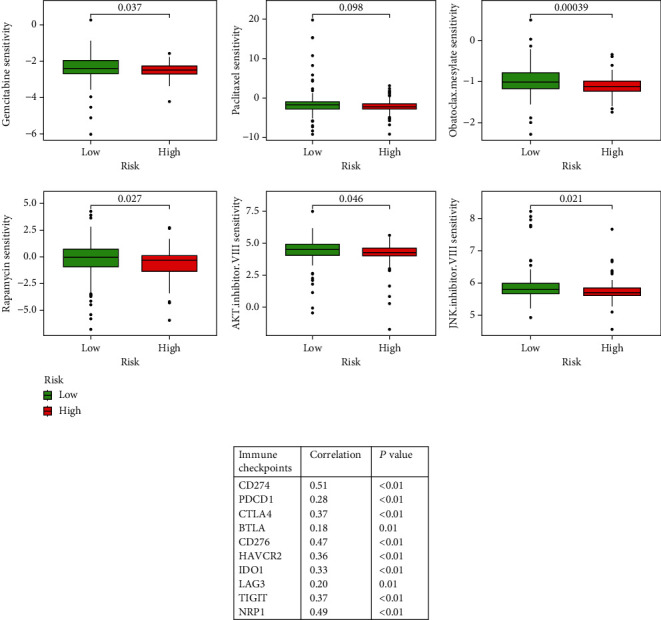
Exploration of the role of brain-derived neurotrophic factor (BDNF) in the medicinal therapy for pancreatic adenocarcinoma (PAAD) patients. (a) Comparisons of the efficacy of common chemotherapeutics in treating PAAD between the low and high BDNF expression subgroups in the Cancer Genome Atlas- (TCGA-) PAAD dataset. (b) Analysis of the correlation between BDNF expression and the expression level of genes related to immune checkpoints in the TCGA-PAAD dataset.

To verify the value of BDNF in clinical treatment for PAAD, we explored the associations between BDNF expression and the efficacy of common administrating chemotherapeutic drugs, and the results revealed that higher BDNF mRNA levels were remarkably correlated with IC50 of chemotherapeutics, such as gemcitabine (*p* = 0.037), rapamycin (*p* = 0.027), obatoclax mesylate (*p* < 0.001), AKT inhibitor VIII (*p* = 0.046), and JNK inhibitor VIII (*p* = 0.021). Although there was no significant difference in the drug sensitivity of paclitaxel between the high BDNF and low BDNF subgroups, IC50 for paclitaxel tended to be reduced with the upregulation of BDNF levels (*p* = 0.098, Figure 8(a)). These findings suggested that high BDNF expression could be predictive of increased chemosensitivity. Since immunotherapy has been recently rapidly developing as a therapeutic renaissance in PAAD, we aimed to determine whether BDNF expression was associated with immune checkpoints and discovered that high BDNF mRNA levels were strongly associated with high expression of CD274, PDCD1, CTLA4, BTLA, CD276, HAVCR2, IDO1, LAG3, TIGIT, and NRP1 (Figure 8(b)).

## 4. Discussion

BDNF is a critical member of the NT polypeptide superfamily, which consists of nerve growth factor (NGF). NGF has been reported to be highly expressed in pancreatic tumors and to be able to promote the migration and invasion of human pancreatic cancer cells [40]. Additionally, trkB, the receptor of BDNF, has been demonstrated to be overexpressed in resected PAAD tissues, which are remarkably associated with perineural invasion and shorter latency to development of liver metastasis in PAAD patients [41]. Although considerable studies have been conducted to understand the biological role of BDNF in various cancers over the past two decades, the functions of BDNF and its utility as a novel biomarker in PAAD have yet to be determined. In the present study, we found that aberrant expression of BDNF was significantly associated with patient prognosis in several types of cancer, with a particularly remarkable association of high BDNF expression with poor prognosis and aggressive clinicopathological characteristics of PAAD patients. Moreover, several cancer-related pathways and the infiltration of immune cells, especially Th2 cells, were significantly correlated with BDNF mRNA levels. Intriguingly, our analyses revealed that the degree of BDNF expression was correlated with high chemotherapeutics sensitivity and highly expressed immune checkpoints, making it a valuable biomarker in predicting the therapeutic benefits for chemotherapy and immunotherapy in cancer patients. To the best of our knowledge, our study provided new insights into understanding the potential role of BDNF in tumor progression and TME of pancreatic cancer.

In the present study, we attempted to profile the pan-cancer expression of BDNF at cell and mRNA level and the association between its abnormal expression and patient outcomes. At the cell detection level, the mRNA expression of BDNF was detected in most of the cancer cell lines, showing an increased tendency when compared to normal tissues. At the mRNA expression level, BDNF expression was downregulated in nine types of tumors compared to normal tissues, whereas others were remarkably elevated in tumor tissues. These discrepant expressions of BDNF revealed that the biological role of BDNF might be context-dependent and serve different functions in different tumors. In the previous study, it still remains unclear whether BDNF can be identified as oncogene or suppressor in different types of cancer. For example, most of the available literature has demonstrated that BDNF can promote tumor development and predict unfavorable prognosis in pan-cancer [14–18]. However, it can also induce cancer remission by eliciting an increased antitumor immune response in multiple cancers [25, 42]. Thus, we performed prognostic analysis of BDNF to define BDNF as tumor oncogene or tumor suppressor, and similar results had been achieved by our study. We found the consistent adverse prognostic value of BDNF expression in PAAD across different databases and BDNF expression in PAAD was positively correlated with tumor T stage, strongly indicating that BDNF served as an unfavorable prognostic biomarker in pancreatic tumors. It was worth noting that BDNF expression levels were remarkably decreased in PAAD patients aged older than 65 years, raising the possibility that elderly patients with pancreatic cancer might represent a heterogeneous subgroup with different molecular characteristics that require individualized antitumor therapy [43].

To better understand the biological functions of BDNF in the regulation of PAAD, functional annotation of BDNF and its similar genes was conducted. Enrichment analysis indicated that these genes were potentially associated with several signaling pathways critical in the tumorigenesis and progression of pancreatic tumors. Among the similar genes, BCL2L1, IRS1, MET, and TGFB2 interacting with BDNF directly was found to be elevated in PAAD tissues. Previous literature revealed that the upregulation of TGFB2 expression can mediate epithelial-mesenchymal transition of pancreatic ductal adenocarcinoma, and MET, IRS1, and BCL2L1 are also demonstrated to be related to PAAD initiation, progression, and metastasis [44–46]. These results suggested that BDNF might exert its tumor-promoting effect through multiple signaling pathways in pancreatic tumor. Moreover, we also evaluated the close association between BDNF expression and some critical pancreatic cancer-promoting genes and found that most of these genes were strongly related to BDNF expression in PAAD, including Akt, mTOR, and MAPK. Previous studies have shown that the phosphoinositide 3-kinase AKT mammalian target of rapamycin (PI3K-AKT-mTOR) signaling pathway exhibits a vital role in the pathogenesis of PAAD and is highly upregulated in pancreatic tumor [47]. RAS-stimulated signaling of MAPK has been reported to be tightly activated in pancreatic cancer, responsible for the initiation and maintenance of pancreatic carcinogenesis [48]. Therefore, we believe that BDNF could regulate PAAD by modulating major oncogenic pathways. In addition, we found that BDNF mRNA levels were associated with several important nodes in apoptosis signaling pathways, including p53, Bcl-1, and Mcl-2. The evasion of apoptosis has been revealed as a novel hallmark of cancer, which can induce tumorigenesis and render tumor cells resistant to multiple therapeutics [49]. In cancer, apoptosis machinery is derived by two canonical approaches: inactivation of the most important tumor suppressor gene p53 and activation of antiapoptotic Bcl-2 family proteins, which comprise Bcl-2 and Mcl-2 [49, 50]. Thus, we hypothesized that BDNF would exert its antiapoptotic property by targeting the two main apoptotic regulators. Intriguingly, there was a highly close association between BDNF and cytidine deaminase (CDA) expression in PAAD based on our correlation analysis. CDA is a cytoplasmic enzyme involved in the metabolism of nucleoside analogs such as gemcitabine, which has long been the backbone of PAAD chemotherapy [3]. The association between CDA expression and the sensitivity/resistance of tumor cells to treatment with gemcitabine is well described [51, 52]. Moreover, severe cellular gemcitabine toxicity has been observed in cancer patients with low activity of CDA [53]. These results demonstrated that BDNF overexpression in PAAD might be another biomarker for determining the resistance to chemotherapy and cellular toxicity.

To the best of our knowledge, this study provides the first evidence of correlation between BDNF and TME in PAAD. Using the ssGSEA method, we found that BDNF expression was positively associated with the abundance of multiple types of T cells in PAAD including central memory CD8+ T cells, central memory CD4+ T cells, and Th2 cells. Neoantigens accumulating on the surface of cancer cells can elicit the antitumor responses through the activation of CD8+ and CD4+ T cells in the TME, subsequently eliminating the tumor cells and thereby preventing cancer development and progression [54]. Different from the conventional view of T cells as a component of anticancer immunity, Th2 cells can help tumor cells escape from the immunosurveillance process by secreting various cytokines [55]. For example, IL6 secreted from Th2 cells plays a critical role in promoting pancreatic cancer development [35]. IL10 produced by Th2 cells is associated with the suppression of the antitumor immune response through inhibiting the antigen processing and presentation of dendritic cells and activating the immunosuppressive regulatory T cells [36, 56]. We found that BDNF was positively related to IL6 and IL10, revealing that BDNF had the potential to modulate the balance of antitumor immune response and immune escape mechanisms in the TME. Besides, Th2 cells showed strong correlation with BDNF expression not only in PAAD but also in multiple cancers. This raised a preliminary assumption that the association between BDNF and Th2 cells might play an important role in tumor immunity. To confirm this notion, we further analyzed the association between BDNF and Th2 cells to illustrate the potential immune-related mechanisms of BDNF in PAAD.

First, a strong association was found between BDNF expression and Th2 cells and their gene markers in the TIMER2.0 database. Furthermore, the levels of Th2 cells were identified to be significantly upregulated in the high BDNF expression group of the TCGA-PAAD cohort. Similar trends were observed in another two independent PAAD cohorts, although there was no statistical significance owing to the restriction of the number of samples and the dynamic changes of tumor immunity during tumor initiation, progression, and metastasis. These findings revealed that Th2 cells had the potential to be activated by BDNF. Second, BDNF was proven to be correlated with chemotaxis in PAAD tissues. Chemokines present at the TME can control the positioning and movements of immune cells. For instance, CCL17 and CCL22, acting on CCR4, can directly recruit Th2 cells at the tumor site, thus participating in the development of cancers [57]. By interacting with CCR2, CCL2 is able to recruit Th2 cells to create an immunosuppression microenvironment [34]. Thus, BDNF might increase the infiltration levels of Th2 cells by regulating the migration of Th2 cells in PAAD. Additionally, the TIMER analysis revealed that Th2 cells predicted worse outcomes in PAAD patients, which was in concordance with the result of a previous study in clear cell renal cell carcinoma and lung squamous cell carcinoma [58, 59]. Finally, we found that there was significant association between Th2 cells and short-term prognosis of PAAD patients under a high expression level of BDNF and between Th2 cells and long-term prognosis under a low expression level of BDNF. We hypothesized that the association between BDNF and Th2 cells might play a tactic role in TME. In the early phase of carcinogenesis, BDNF might promote the growth of cancer by activating Th2 cells and therefore affect the survival of PAAD patients. Once a tumor progresses past this early stage, Th2 cells gradually played a predominant role in supporting pancreatic cancer cells. Future mechanistic investigation focusing on BDNF expression and Th2 cell infiltration in the TME of PAAD is needed to prove this assumption. The above data suggested that BDNF might be involved in the immune response via interacting with Th2 cells, contributing to pancreatic carcinogenesis and progression, thus resulting in an unfavorable prognosis of PAAD patients.

Another important finding of this study was the significance of BDNF in the clinical treatment for PAAD. As is well known, tumor-infiltrating immune cells can affect the response to immunotherapy and chemotherapy [11, 12]. Since BDNF was proven to be significantly associated with immune cells in PAAD, we elaborated the association between BDNF expression and the immunosuppressive molecules expressing immune checkpoint inhibitors (ICIs) and found that BDNF was positively correlated with ICI-related biomarkers. Thus, we suspected that combining BDNF blockade and ICIs might be a feasible approach to eliminate cancer cells. Furthermore, our study demonstrated that BDNF was associated with sensitivity to several chemotherapeutic drugs, which are recommended for PAAD treatment by the AJCC guidelines. These results revealed that BDNF might be a potential biomarker for predicting the efficacy of chemotherapeutics and ICIs for PAAD treatment. Although most studies identified BDNF as a universal attenuator of chemotherapeutic efficacy in vitro [60, 61], the role of BDNF in modulating the resistance to chemotherapy in vivo remains largely unknown. Thus, prospective clinical studies are required.

Despite some strengths of the current study, it has some limitations. First, our study assessing the association between BDNF and PAAD was conducted based on information extracted from open-access databases, and no experimental validation was performed to confirm these predicted results. Second, exploration of BDNF expression was based entirely on the mRNA levels reported in the TCGA, GEO, GTEx, and CCLE databases. However, this could not predict protein expression and reflect posttranslational modification of BDNF. Further studies should pay attention to validate the protein levels of BDNF in PAAD using immunohistochemistry, immunocytochemistry, or immunoblotting. Finally, despite our analyses revealing that the mRNA levels of BDNF were significantly associated with Th2 cell infiltration and patient prognosis in PAAD, we still could not conclude that BDNF directly influenced patient outcomes by activating Th2 cells. Additionally, whether the therapy patients have received in the current study could influence the association of BDNF and immune cell infiltration or not remained unclear. In vivo/in vitro mechanistic investigation and even clinical trials should be conducted to clarify the underlying mechanism.

## 5. Conclusion

To the best of our knowledge, this was the first study to depict the potential functions of BDNF in tumor immunity and its predicted value in pancreatic cancer by applying integrated bioinformatics approaches. BDNF might affect patient prognosis by mediating the infiltration levels of Th2 cells, providing a novel direction to explore the pathogenesis and malignancy of PAAD. Our study also revealed that BDNF was positively correlated with chemotherapeutics efficacy and immunosuppressed biomarkers, suggesting that BDNF is an antitumor target in pancreatic cancer.

## Figures and Tables

**Table 1:** Correlation between BDNF expression and chemokines in PAAD.

Chemokine	Correlation	*p* value
CCL1	−0.046	0.542
CCL2	0.348	<0.001
CCL3	0.180	0.016
CCL4	0.277	<0.001
CCL5	0.317	<0.001
CCL7	0.370	<0.001
CCL8	0.290	<0.001
CCL11	0.432	<0.001
CCL13	0.309	<0.001
CCL14	0.012	0.874
CCL15	−0.161	0.032
CCL16	−0.134	0.075
CCL17	0.223	0.003
CCL18	0.299	<0.001
CCL19	0.194	0.009
CCL20	0.230	0.002
CCL21	0.139	0.064
CCL22	0.317	<0.001
CCL23	0.081	0.280
CCL24	−0.034	0.648
CCL25	−0.007	0.927
CCL26	0.053	0.479
CCL27	0.047	0.533
CCL28	0.327	<0.001
CX3CL1	0.373	<0.001

**Table 2:** Correlation between BDNF expression and chemokine receptors in PAAD.

Chemokine receptor	Correlation	*p* value
CCR1	0.409	<0.001
CCR2	0.281	<0.001
CCR3	0.334	<0.001
CCR4	0.386	<0.001
CCR5	0.335	<0.001
CCR6	0.131	0.081
CCR7	0.263	<0.001
CCR8	0.408	<0.001
CCR9	0.291	<0.001
CCR10	0.077	0.304
CX3CR1	0.073	0.334
CXCR1	0.181	0.016
CXCR2	0.319	<0.001
CXCR3	0.305	<0.001
CXCR4	0.264	<0.001
CXCR5	0.260	<0.001
CXCR6	0.320	<0.001
XCR1	0.249	0.001

## Data Availability

The datasets generated for this study can be found in online repositories. The names of the repository/repositories and accession number(s) can be found in the article.

## Supplementary Materials

Supplementary Materials Supplementary Tables include the following information. Table S1: summary of TCGA cancer types. Table S2: top 100 similar genes associated with BDNF in PAAD. Table S3: KEGG enrichment analysis of BDNF and its similar genes. Table S4: GO enrichment analysis of BDNF and its similar genes.
