# Supplementary material for: BDNF Acts as a Prognostic Factor Associated with Tumor-Infiltrating Th2 Cells in Pancreatic Adenocarcinoma
Source: Dis Markers. 2021 Nov 5;2021:7842035. doi: 10.1155/2021/7842035 (PMC8589485; doi:10.1155/2021/7842035)
Supplement: Supplementary Materials — Supplementary Tables include the following information. Table S1: summary of TCGA cancer types. Table S2: top 100 similar genes associated with BDNF in PAAD. Table S3: KEGG enrichment analysis of BDNF and its similar genes. Table S4: GO enrichment analysis of BDNF and its similar genes. [file 7842035.f1.doc]

**Table S1 Summary of TCGA cancer types.**

| **TCGA Cancer Abbreviation** | **TCGA Cancer Type** |
| --- | --- |
| **ACC** | Adrenocortical carcinoma |
| **BLCA** | Bladder Urothelial Carcinoma |
| **LGG** | Brain Lower Grade Glioma |
| **BRCA** | Breast invasive carcinoma |
| **CESC** | Cervical squamous cell carcinoma and endocervical adenocarcinoma |
| **CHOL** | Cholangiocarcinoma |
| **COAD** | Colon adenocarcinoma |
| **ESCA** | Esophageal carcinoma |
| **GBM** | Glioblastoma multiforme |
| **HNSC** | Head and Neck squamous cell carcinoma |
| **KICH** | Kidney Chromophobe |
| **KIRC** | Kidney renal clear cell carcinoma |
| **KIRP** | Kidney renal papillary cell carcinoma |
| **LIHC** | Liver hepatocellular carcinoma |
| **LUAD** | Lung adenocarcinoma |
| **LUSC** | Lung squamous cell carcinoma |
| **LAML** | Acute Myeloid Leukemia |
| **MESO** | Mesothelioma |
| **OV** | Ovarian serous cystadenocarcinoma |
| **PAAD** | Pancreatic adenocarcinoma |
| **PRAD** | Prostate adenocarcinoma |
| **READ** | Rectum adenocarcinoma |
| **SKCM** | Skin Cutaneous Melanoma |
| **STAD** | Stomach adenocarcinoma |
| **TGCT** | Testicular Germ Cell Tumors |
| **THCA** | Thyroid carcinoma |
| **UCS** | Uterine Carcinosarcoma |
| **UCEC** | Uterine Corpus Endometrial Carcinoma |

**Table S2 Top 100 similar genes associated with BDNF in PAAD.**

| **Gene Symbol** | **Gene ID** | **Pearson correlation coefficient** |
| --- | --- | --- |
| **RP11-115J23.1** | ENSG00000249867.5 | 0.66 |
| **ABALON** | ENSG00000281376.1 | 0.57 |
| **RND3** | ENSG00000115963.13 | 0.54 |
| **KIF18A** | ENSG00000121621.6 | 0.52 |
| **PRKG1-AS1** | ENSG00000236671.7 | 0.51 |
| **TGFB2** | ENSG00000092969.11 | 0.5 |
| **TGFA** | ENSG00000163235.15 | 0.5 |
| **DKK1** | ENSG00000107984.9 | 0.5 |
| **RBMS2** | ENSG00000076067.11 | 0.5 |
| **EHBP1** | ENSG00000115504.14 | 0.5 |
| **TGFB2-OT1** | ENSG00000281453.1 | 0.49 |
| **BCL2L1** | ENSG00000171552.12 | 0.49 |
| **TMEM133** | ENSG00000170647.3 | 0.48 |
| **RNASE9** | ENSG00000188655.10 | 0.47 |
| **AJUBA** | ENSG00000129474.15 | 0.47 |
| **RP11-54H7.4** | ENSG00000275216.1 | 0.47 |
| **ITGB8** | ENSG00000105855.9 | 0.46 |
| **HRH1** | ENSG00000196639.6 | 0.46 |
| **TMEM169** | ENSG00000163449.10 | 0.45 |
| **IRS1** | ENSG00000169047.5 | 0.45 |
| **SPAG1** | ENSG00000104450.12 | 0.45 |
| **PMAIP1** | ENSG00000141682.11 | 0.45 |
| **NUDCD1** | ENSG00000120526.10 | 0.45 |
| **UPK2** | ENSG00000110375.2 | 0.45 |
| **ELK3** | ENSG00000111145.7 | 0.44 |
| **MET** | ENSG00000105976.14 | 0.44 |
| **PSMC3** | ENSG00000165916.8 | 0.43 |
| **WNT7B** | ENSG00000188064.9 | 0.43 |
| **RP11-722M1.1** | ENSG00000248215.5 | 0.43 |
| **INPP4B** | ENSG00000109452.12 | 0.43 |
| **MIR4435-2HG** | ENSG00000172965.14 | 0.42 |
| **SET** | ENSG00000119335.16 | 0.42 |
| **RP11-161I2.1** | ENSG00000187229.3 | 0.42 |
| **RP11-395N3.1** | ENSG00000261379.1 | 0.42 |
| **CSH2** | ENSG00000213218.9 | 0.42 |
| **NEK7** | ENSG00000151414.14 | 0.42 |
| **DCBLD2** | ENSG00000057019.15 | 0.42 |
| **RN7SL419P** | ENSG00000243883.3 | 0.42 |
| **TPM4** | ENSG00000167460.14 | 0.42 |
| **RP11-479G22.8** | ENSG00000273038.2 | 0.42 |
| **TUBB** | ENSG00000196230.12 | 0.42 |
| **AXL** | ENSG00000167601.11 | 0.42 |
| **AMOTL2** | ENSG00000114019.14 | 0.41 |
| **RP11-791N19.1** | ENSG00000279682.1 | 0.41 |
| **LYPD5** | ENSG00000159871.14 | 0.41 |
| **NEMP2** | ENSG00000189362.11 | 0.41 |
| **ITGB1** | ENSG00000150093.18 | 0.41 |
| **OR7E149P** | ENSG00000177586.6 | 0.41 |
| **TNFAIP1** | ENSG00000109079.9 | 0.41 |
| **KRT18P60** | ENSG00000215208.3 | 0.41 |
| **ARHGAP23** | ENSG00000275832.4 | 0.41 |
| **LLPH** | ENSG00000139233.6 | 0.41 |
| **ARL6IP6** | ENSG00000177917.10 | 0.41 |
| **ANLN** | ENSG00000011426.10 | 0.41 |
| **TCAM1P** | ENSG00000240280.6 | 0.41 |
| **SSRP1** | ENSG00000149136.7 | 0.41 |
| **RP4-781K5.9** | ENSG00000272362.1 | 0.40 |
| **NR2F1-AS1** | ENSG00000237187.8 | 0.40 |
| **HTRA2** | ENSG00000115317.11 | 0.40 |
| **NET1** | ENSG00000173848.18 | 0.40 |
| **RARRES3** | ENSG00000133321.10 | 0.40 |
| **Metazoa_SRP** | ENSG00000273866.1 | 0.40 |
| **CDK2** | ENSG00000123374.10 | 0.40 |
| **FZD6** | ENSG00000164930.11 | 0.40 |
| **UHRF1** | ENSG00000276043.4 | 0.40 |
| **ARL4D** | ENSG00000175906.4 | 0.40 |
| **TCEA1** | ENSG00000187735.12 | 0.40 |
| **BACH1** | ENSG00000156273.15 | 0.40 |
| **CHEK1** | ENSG00000149554.12 | 0.40 |
| **RP11-49K24.4** | ENSG00000266957.1 | 0.40 |
| **HMGA1P1** | ENSG00000236683.3 | 0.40 |
| **PLA2G16** | ENSG00000176485.10 | 0.40 |
| **SPDL1** | ENSG00000040275.16 | 0.4 |
| **ARHGAP42** | ENSG00000165895.17 | 0.4 |
| **DUSP14** | ENSG00000276023.4 | 0.4 |
| **RP11-1100L3.4** | ENSG00000258021.1 | 0.4 |
| **SLC20A2** | ENSG00000168575.9 | 0.4 |
| **CCDC81** | ENSG00000149201.9 | 0.4 |
| **ITGB1BP1** | ENSG00000119185.12 | 0.4 |
| **NRP2** | ENSG00000118257.16 | 0.39 |
| **SKA2** | ENSG00000182628.12 | 0.39 |
| **ISY1** | ENSG00000240682.9 | 0.39 |
| **IKZF2** | ENSG00000030419.16 | 0.39 |
| **FLII** | ENSG00000177731.15 | 0.39 |
| **TES** | ENSG00000135269.17 | 0.39 |
| **RP11-204C16.4** | ENSG00000217624.2 | 0.39 |
| **BAG4** | ENSG00000156735.10 | 0.39 |
| **TRAM1** | ENSG00000067167.7 | 0.39 |
| **DDX27** | ENSG00000124228.14 | 0.39 |
| **STIP1** | ENSG00000168439.16 | 0.39 |
| **SMURF2** | ENSG00000108854.15 | 0.39 |
| **TPD52L2** | ENSG00000101150.17 | 0.39 |
| **LLGL1** | ENSG00000131899.10 | 0.39 |
| **TMOD3** | ENSG00000138594.12 | 0.39 |
| **VGLL1** | ENSG00000102243.12 | 0.39 |
| **C18orf54** | ENSG00000166845.13 | 0.39 |
| **ZPR1** | ENSG00000109917.10 | 0.39 |
| **PRRG1** | ENSG00000130962.17 | 0.39 |
| **GTDC1** | ENSG00000121964.14 | 0.39 |

**Table S3 KEGG enrichment analysis of BDNF and its similar genes.**

| **ID** | **Description** | **p-value** | **q-value** | **Count** |
| --- | --- | --- | --- | --- |
| **hsa05225** | Hepatocellular carcinoma | <0.001 | 0.001 | 6 |
| **hsa04151** | PI3K-Akt signaling pathway | <0.001 | 0.001 | 8 |
| **hsa04115** | p53 signaling pathway | <0.001 | 0.003 | 4 |
| **hsa01521** | EGFR tyrosine kinase inhibitor resistance | <0.001 | 0.003 | 4 |
| **hsa05226** | Gastric cancer | <0.001 | 0.003 | 5 |
| **hsa04215** | Apoptosis - multiple species | <0.001 | 0.003 | 3 |
| **hsa04390** | Hippo signaling pathway | <0.001 | 0.003 | 5 |
| **hsa05410** | Hypertrophic cardiomyopathy | <0.001 | 0.003 | 4 |
| **hsa05414** | Dilated cardiomyopathy | <0.001 | 0.004 | 4 |
| **hsa05205** | Proteoglycans in cancer | 0.001 | 0.007 | 5 |
| **hsa05165** | Human papillomavirus infection | 0.001 | 0.009 | 6 |
| **hsa05022** | Pathways of neurodegeneration - multiple diseases | 0.001 | 0.010 | 7 |
| **hsa05010** | Alzheimer disease | 0.002 | 0.012 | 6 |
| **hsa05211** | Renal cell carcinoma | 0.002 | 0.012 | 3 |
| **hsa05212** | Pancreatic cancer | 0.003 | 0.015 | 3 |
| **hsa05210** | Colorectal cancer | 0.004 | 0.020 | 3 |
| **hsa05222** | Small cell lung cancer | 0.005 | 0.023 | 3 |

**Table S4 GO enrichment analysis of BDNF and its similar genes.**

| **ID** | **Description** | **p-value** | **q-value** | **Count** |
| --- | --- | --- | --- | --- |
| **GO:0031032** | actomyosin structure organization | <0.001 | 0.001 | 8 |
| **GO:0035024** | negative regulation of Rho protein signal transduction | <0.001 | 0.001 | 4 |
| **GO:0046580** | negative regulation of Ras protein signal transduction | <0.001 | 0.001 | 5 |
| **GO:0051058** | negative regulation of small GTPase mediated signal transduction | <0.001 | 0.001 | 5 |
| **GO:0051897** | positive regulation of protein kinase B signaling | <0.001 | 0.001 | 7 |
| **GO:0035023** | regulation of Rho protein signal transduction | <0.001 | 0.003 | 5 |
| **GO:0010639** | negative regulation of organelle organization | <0.001 | 0.003 | 9 |
| **GO:0051896** | regulation of protein kinase B signaling | <0.001 | 0.006 | 7 |
| **GO:0001667** | ameboidal-type cell migration | <0.001 | 0.008 | 9 |
| **GO:0043491** | protein kinase B signaling | <0.001 | 0.008 | 7 |
| **GO:0046578** | regulation of Ras protein signal transduction | <0.001 | 0.009 | 6 |
| **GO:0033627** | cell adhesion mediated by integrin | <0.001 | 0.012 | 4 |
| **GO:0007163** | establishment or maintenance of cell polarity | <0.001 | 0.012 | 6 |
| **GO:0007266** | Rho protein signal transduction | <0.001 | 0.012 | 5 |
| **GO:1901214** | regulation of neuron death | <0.001 | 0.012 | 7 |
| **GO:0051056** | regulation of small GTPase mediated signal transduction | <0.001 | 0.012 | 7 |
| **GO:0007015** | actin filament organization | <0.001 | 0.012 | 8 |
| **GO:0007265** | Ras protein signal transduction | <0.001 | 0.018 | 7 |
| **GO:0010762** | regulation of fibroblast migration | <0.001 | 0.018 | 3 |
| **GO:0051294** | establishment of spindle orientation | <0.001 | 0.019 | 3 |
| **GO:0070997** | neuron death | <0.001 | 0.019 | 7 |
| **GO:0006260** | DNA replication | <0.001 | 0.026 | 6 |
| **GO:0044839** | cell cycle G2/M phase transition | <0.001 | 0.026 | 6 |
| **GO:0051293** | establishment of spindle localization | 0.001 | 0.030 | 3 |
| **GO:0030038** | contractile actin filament bundle assembly | 0.001 | 0.030 | 4 |
| **GO:0043149** | stress fiber assembly | 0.001 | 0.030 | 4 |
| **GO:0010761** | fibroblast migration | 0.001 | 0.030 | 3 |
| **GO:0060071** | Wnt signaling pathway, planar cell polarity pathway | 0.001 | 0.030 | 4 |
| **GO:0090175** | regulation of establishment of planar polarity | 0.001 | 0.031 | 4 |
| **GO:0051653** | spindle localization | 0.001 | 0.036 | 3 |
